# RecSOI: recommending research directions using statements of ignorance

**DOI:** 10.1186/s13326-024-00304-3

**Published:** 2024-04-22

**Authors:** Adrien Bibal, Nourah M. Salem, Rémi Cardon, Elizabeth K. White, Daniel E. Acuna, Robin Burke, Lawrence E. Hunter

**Affiliations:** 1 University of Colorado Anschutz Medical Campus, Aurora, Colorado USA; 2 University of Louvain, Louvain-la-Neuve, Belgium; 3 University of Colorado Boulder, Boulder, Colorado USA; 4 University of Chicago, Chicago, Illinois USA

**Keywords:** Recommender systems, Biomedical literature, Natural Language Processing, NLP, Science of science, Statements of ignorance

## Abstract

The more science advances, the more questions are asked. This compounding growth can make it difficult to keep up with current research directions. Furthermore, this difficulty is exacerbated for junior researchers who enter fields with already large bases of potentially fruitful research avenues. In this paper, we propose a novel task and a recommender system for research directions, RecSOI, that draws from statements of ignorance (SOIs) found in the research literature. By building researchers’ profiles based on textual elements, RecSOI generates personalized recommendations of potential research directions tailored to their interests. In addition, RecSOI provides context for the recommended SOIs, so that users can quickly evaluate how relevant the research direction is for them. In this paper, we provide an overview of RecSOI’s functioning, implementation, and evaluation, demonstrating its effectiveness in guiding researchers through the vast landscape of potential research directions.

## Background

Finding new research topics is a task that researchers must handle very often, especially when starting a PhD degree. However, navigating the increasingly vast expanse of scientific knowledge, which sees a doubling of publication output every 17.3 years [1], is an arduous task for even the most experienced academics. Amid the many papers published each year and the surge of scientists joining the workforce, pinpointing the most suitable research direction becomes increasingly challenging. Some argue that this phenomenon could be one of the reasons behind the seeming slowdown of novel scientific progress [2–4]. This observation underlines the importance of managing the vast and rapidly increasing volume of existing knowledge and being able to discern gaps and opportunities for innovation. It stands to reason that researchers in science, and especially newcomers, would therefore benefit from a recommender system that provides them with research directions that align with their profile or the profiles of their collaborators or their supervisor. While this paper focuses on helping new researcher find research directions that are relevant to them, many other use cases exist for our recommender system (see, e.g., Boguslav et al. [5] for some ideas of use cases). For instance, another use case could be to help principal investigators (PIs) navigate the literature in order to find the crucial state-of-the-art problems that match the expertise of their lab. Not only would this help PIs target suitable grant funding, but it would also help society, as difficult problems would be matched to researchers with the corresponding skills.

**Fig. 1:**
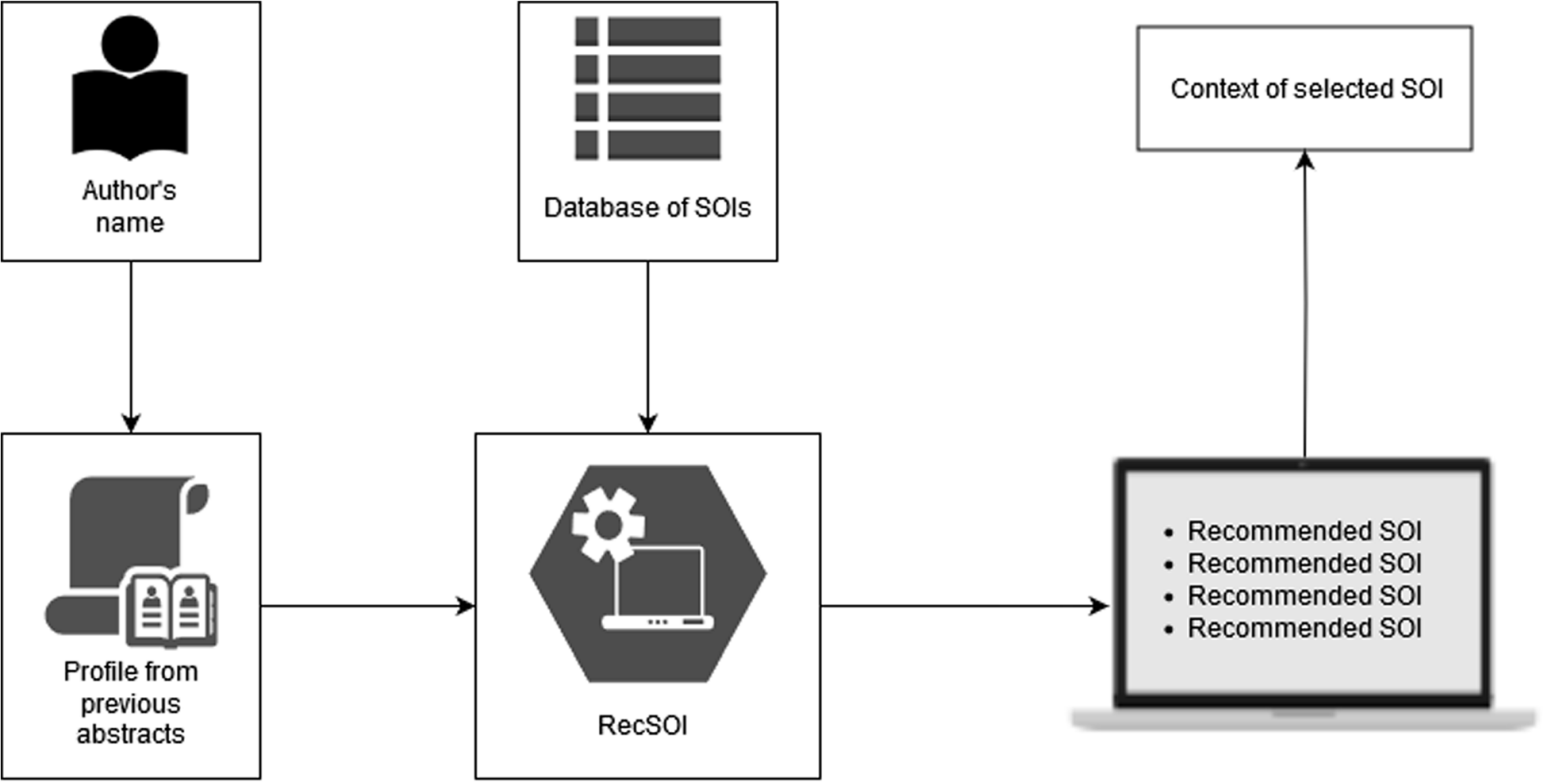
RecSOI pipeline, from an author name provided as input to a list of recommended directions and their context

Such a recommender system is only possible if new research directions can be extracted from papers in the literature. In order to accomplish that, Boguslav et al. [5, 6] recently provided ways to identify sentences in papers stating a lack of knowledge, or ignorance, that can then be used to discover possible research directions. Therefore, starting from the premise that identifying such statements of ignorance (SOIs) is possible, we propose a novel task and a new system, RecSOI (Recommender of research directions using Statements Of Ignorance), to recommend to researchers, based on their profile, SOIs that they would be interested in investigating. Furthermore, RecSOI’s pipeline provides a module for extracting the SOI’s context from the paper. With this background information, researchers may be able to get the gist of most recommended research directions without needing to read the papers that mention them. A user evaluation is proposed in this paper to make it possible to assess the importance of extracting context. The overall RecSOI pipeline can be seen in Fig. 1. Our main contributions are the following:

- A description of a way to recommend research directions based on statements of ignorance in papers;
- An estimation of the difficulty of the task;
- A system, called RecSOI, for recommending research directions to researchers;
- A user evaluation of the context that can be provided alongside recommended research directions;
- A detailed discussion about the task, including about potential fairness issues.

In order to introduce our novel task and RecSOI, we first start by providing some work related to ours in the Related work section. Then, we provide some background about statements of ignorance in the Statements of ignorance section. We introduce the problem of identifying relevant research directions from these statements of ignorance in the Research directions using SOIs section. RecSOI and its evaluation are then presented in the Methods section. The results of our evaluation are presented in the Results section. An analysis of the extraction of context to better understand the recommendations is provided in the Extracting ignorance context section. We close the paper with a detailed discussion in the Discussion section and our conclusion in the Conclusion section.

### Related work

Our work is closely related to Boguslav et al.’s [5, 6], which detects SOIs. However, we extend that work by recommending new research directions based on these SOIs to researchers.

There are many components of scientific discourse that can help us navigate the current state of knowledge. One such type is claims, which are related to finding the current knowledge, or answers, in the literature. Achakulvisut et al. [7] propose to extract scientific *claims* from the literature. Another type of discourse is *arguments*, which are logical and evidence-based processes that seek to establish or support a particular scientific claim or hypothesis. The work of Stab et al. [8] aims to develop argument-mining methods in the context of scientific papers and persuasive essays and draws conclusions about these tasks. Another type, and the focus of our paper, concerns discourses about the *known unknowns*, which Boguslav et al. [5] call statements of ignorance (SOIs). In contrast to claims and arguments, these SOIs have been much less studied.

Close to our work, Lahav et al. [9] propose a search engine for research directions given a certain topic (e.g., COVID-19). Like Boguslav et al. [5], who identify sentences with SOIs, Lahav et al. identify sentences that contain mentions of challenges or research directions and then index these sentences based on the entities contained in them. One of the main differences between these two works is that Boguslav et al. focus on analyzing, describing and categorizing the SOIs, while Lahav et al. focus on providing a search engine on top of the detected challenges/directions. Like Lahav et al., we focus on recommending research directions, but our reliance on researcher profiles, rather than keywords, allows us to tailor searches to the interests and expertise of the researcher, so that, for example, instead of finding new research directions related to “COVID-19”, we can provide directions that are more specific to the interests of the researcher (e.g., “the impact of COVID-19 on the heart”).

The field of recommender systems is rich with studies aiming to select research papers from various perspectives (for more information, see Bai et al. [10]), and many papers in the literature tackle this problem, each through a different lens (see, e.g., [11–22]). Our work is unique in recommending research directions mentioned in these research papers. Although one can argue that by recommending research directions, we are also implicitly recommending papers from the literature (i.e., the papers that contain the directions), we also justify why each paper is recommended based on the potentially relevant research directions that it mentions. This innovation takes us a step beyond traditional paper recommendation to create a richer, more helpful guidance system for researchers.

### Statements of ignorance

Boguslav et al. recently developed the term “statements of ignorance” [5] (SOIs), inspired by the work of Firestein [23], and defined it as “statements about knowledge that does not exist yet” [5]. In their work, Boguslav et al. thoroughly studied the concept by identifying the different categories of SOIs in the literature. The authors also analyzed the different lexical cues that are often present in each category of SOIs.

In this work, we are interested in the SOIs that can indicate a possible new research direction for a researcher. Indeed, if a paper states that something is still unknown and deserves more investigation, then this lead can probably be used to start a new research project. This, therefore, means that we are interested in the subset of the SOI categories that indicate possible new research directions. All of the categories highlighted by Boguslav et al. [5] (see Table 1) pertain to a lack of knowledge. Some categories are related to how the lack of knowledge is expressed (e.g., “explicit questions” and “future work”). Other categories relate to the intent of the statements (e.g., “question answered by this work” serves the purpose of motivating the paper stating it). The SOI categories that are relevant for our recommendation of research directions are “full unknown”, “explicit question”, “problem or complication”, “future work” and “future prediction”.

**Table 1:** Table inspired by the first table of Boguslav et al. [5], showing different categories of statements of ignorance

Categories	Short explanation
Question answered by this work	Statement about an ignorance stated in the work and answered in the same work
Full unknown	Statement with an explicit mention of something that is not known
Explicit question	Explicit question signaling a lack of knowledge
Incomplete evidence	(1) Proposed explanation/hypothesis grounded on incomplete evidence, or (2) statement about a lack of evidence
Superficial relationship	Statement noting the presence of a relationship between two or more variables
Probable understanding	Statement about an uncertain, but seemingly likely correct, understanding
Anomaly/curious finding	Statement about an unexpected result or finding
Alternative options/controversy	Statement about different points of views on a subject, including a potential controversy
Difficult task	Statement mentioning that something is difficult to accomplish
Problem/complication	Statement about problems or complications in the study
Future work	Statement about next steps to accomplish
Future prediction	Extrapolation based on actual data/information
Important consideration	Statement pointing to an urgent matter to consider

### Research directions using SOIs

As SOIs state a certain lack of knowledge, one can investigate this lack of knowledge to pursue new research directions. For instance, a sentence that mentions that “the relation between X and Y is unexpected and requires further investigations” indicates that a new research direction would be to investigate this relation between X and Y more deeply.

However, one issue with SOIs, which Boguslav et al. [5] found in their study, is that many sentences in papers can be considered to be SOIs. In fact, as we have also discovered in our research, approximately half of the sentences in papers can be perceived to contain a certain form of ignorance. As a consequence, parsing all papers of the literature (or of a certain field of the literature) and extracting the SOIs would leave researchers with hundreds of thousands, if not millions, of SOIs to explore - an impractical number for researchers to leverage them for finding new research directions.

This motivates the need to build a recommender system on top of these SOIs in order to rank them with respect to the researcher’s interests and expertise. This entails (1) building a researcher profile that can be used for recommendation, (2) vectorizing the SOIs in the database to make them candidates for a recommendation system, and (3) linking researcher profiles to relevant research directions for them. Please note that for (3), we make the assumption that research directions are more interesting for a researcher if they relate to research subjects that are close to the researcher’s own work. One can argue that some researchers may be interested in research directions that deviate from their own work. We leave the recommendation of this kind of research directions as a future work. In the next section, we show how RecSOI, our proposed method, can recommend new research directions to researchers, leveraging a database of SOIs.

## Methods

This section introduces RecSOI through two steps: building the profile of the researcher and then recommending research directions based on that profile.

### Researcher profile embedding

The first step to recommending research directions is summarizing researchers’ work in a certain vector, or embedding, space. Two strategies can be used to summarize researcher profiles. First, to achieve good recommendation performance, it can be important to consider specific combinations of concepts that are often invoked by the researcher, rather than full texts. This is not effective for all researchers, as it ignores the big picture. Second, having a comprehensive view of what and how the researcher wrote can also be important for knowing what to recommend. However, this strategy is weaker when important concepts are buried in many irrelevant texts. Although these two strategies tend to work for different subsets of researchers, neither one provides a “one size fits all” solution.

In our recommender system of statements of ignorance (RecSOI), we propose in this section to combine the best of the two worlds to embed researcher profiles. For a particular abstract *a* (without the title of the article) from researcher *r*, a sentence-BERT model [24] (more precisely, “sentence-transformers/all-MiniLM-L6-v2”) is first run on each sentence $s_i$ of this abstract to obtain the corresponding embedding $e_i$.

Different versions of BERT (like, e.g., BioBERT [25]) have been tested in our preliminary experiments. sentence-BERT was chosen has the embedding models for four reasons. First, we observed during our preliminary experiments that the recommendation results were not better when using more specific versions of BERT. Second, due to the sensitivity of our recommender to overfitting (see the Discussion section for a discussion about that), we decided to opt for the most generic version of BERT. Third, by not using very specific versions of BERT like BioBERT and BlueBERT [26], we also want to show that the task can be extended to other fields than the biomedical field. Finally, as our most important elements (SOIs) are expressed with sentences, we decided to work with a version of BERT that is fine-tuned to embed sentences.

For the next step, a logistic regression model (LR) is run on the same sentences to get the probability that the sentence was written by this author. In order to do so, LR is trained on a dataset of abstracts in a binary classification setup: 1 if the first author of the abstract is *r* and 0 otherwise. Then, in a similar fashion as the Rocchio algorithm [27], the final embedding of the abstract *a* is the average of the embedding of the sentences in *a*, positively or negatively weighted by LR predictions. The LR predictions are therefore used to estimate the relevance of the sentences in the average. The objective is to obtain a representation of the abstract in the embedding space that is as close as possible to the sentences that are representative to the author. More formally, the weight for each sentence $s_i$ is given by

$$\begin{aligned} \text {weight}(s_i) = \left\{ \begin{array}{ll} -(2*(0.5 - \text {LR}(s_i))), &{} \text {if LR}(s_i) < 0.5\\ (2*(\text {LR}(s_i) - 0.5)), &{} \text {otherwise,} \end{array}\right. \end{aligned}$$

with LR$(s_i)$ being a probability given by the LR prediction for $s_i$ when a TF-IDF vectorization of $s_i$ is considered. The use of a TF-IDF vectorization of the researcher’s papers allows to put an emphasis on the concepts that are specifically used by the researcher. The abstract embedding(*a*) is then given by

$$\begin{aligned} \text {embedding}(a) = \frac{\sum \nolimits _{s_i \in a} \text {weight}(s_i)*e_i}{\sum \nolimits _{s_i \in a} |\text {weight}(s_i)|}, \end{aligned}$$

where $e_i$ is the sentence-BERT embedding of $s_i$ and $|\text {weight}(s_i)|$ is the absolute value of $\text {weight}(s_i)$.

The final profile of researcher *r* is then built by keeping a list of each $\text {embedding}(a_j)$ for all available abstracts $a_j$ of researcher *r*. In our case, as we consider the use case in which junior researchers look for research directions, the number of abstracts for each researcher is between 1 and 5.

As our profile embedding is designed to work on previous abstracts, very new researchers (i.e. with no previous papers) may face limitations to use our system. Three solutions can help bootstrap the system in such a case. In the first solution, if the new researcher has at least one paper but is not first author for any of them, these papers can be used if they are close enough to the general research direction the new researcher will take in their main research. In the second solution, one can use the profile of another researcher with similar research interests (e.g., their supervisor, or a PhD student working on a similar question). In the third solution, one can use methods relying on keywords, such as the one of Lahav et al. [9], until at least one abstract is available to build a profile.

### Recommending research directions

During our preliminary experiments, we found that using metric learning methods to learn the best distance between user profiles and SOIs did not perform well. Based on other results discussed later in this paper, our assumption is that building such a metric learning model has the tendency to overfit in our task. On the other hand, classic metrics like the Euclidean distance and the cosine similarity perform quite well. As overcomplicating the solution tends to provide a lower performance (because of the overfitting effect), our best solution was to simply compute the Euclidean distance between the author profile and each SOI candidate. The candidates that are then recommended are the ones for which the distance to at least one abstract in the author’s profile is the smallest.

Note that one important advantage of RecSOI is that given the definition of the profile embedding in the Researcher profile embedding section and the use of an Euclidean distance for matching profiles to research directions, RecSOI is not dependent on numerical hyperparameters to tune. The only components of RecSOI that can be investigated and improved in future work are (1) the model used to weight the abstract’s sentences in the user profiles, and (2) the distance measure between the user profiles and the research directions. The choices made in this paper for these components are the ones that provided the best results during our preliminary experiments. Another interesting feature of the recommendations of RecSOI is that they are deterministic, which means that, for a given user profile and a given database of SOIs, the recommendations will always be the same.

### Evaluation

In order to evaluate RecSOI, we propose a quantitative experiment followed by a qualitative analysis of the errors to better understand the results. In the quantitative evaluation, three heuristics are used to assess the quality of the recommendations. In the qualitative evaluation, particular SOIs are studied to better understand the difficulty of the problem.

#### Experimental setup

In order to explain our experimental setup, three elements need to be presented. First, we base our evaluation on a uniquely annotated dataset from the biomedical literature, but we needed to expand it further. The dataset and the process used to augment it are described in the Dataset section. Then, as it is not realistic to gather experts to evaluate 500 recommendations from a very specific field of science, three heuristics are proposed in the Evaluation heuristics section to assess the quality of the recommendation. Finally, we present in the Baseline methods section the baseline methods we use to compare to RecSOI.

#### Dataset

Boguslav et al. developed classifiers with a high performance for classifying whether a sentence is a SOI or not [5]. The testing F1-score that they report is 0.85 when the positive class contains SOIs of all categories and the negative class contains the other regular sentences [5]. These classifiers came alongside a dataset of papers on prenatal nutrition. This dataset is the only dataset in the literature that contains hand-crafted annotations about SOIs and their category. Indeed, the main feature of this dataset is that it went through a thorough annotation campaign with experts in the domain of prenatal nutrition. During the annotation campaign, the sentences containing a certain lack of knowledge were annotated alongside their corresponding category of ignorance.

For our study, we consider Boguslav et al.’s dataset of SOIs as potential research directions because of its unique expert annotations and the well-performing classifiers [5, 6]. However, for three reasons, we needed to extend Boguslav et al.’s dataset to make the evaluation of our recommendations possible. Indeed, (1) there were only 60 papers in the dataset, (2) each first author in the dataset has only one paper as first author, and (3) there are no previous papers, abstracts or other information provided in Boguslav et al.’s dataset, which would be used to build a profile for each author and make recommendations based on it.

In order to augment Boguslav et al.’s dataset, we proceed in three steps. In the first step, we gather the PubMed ID (PMID) of the 10,000 papers that are the closest to the “prenatal nutrition” subject using the PubMed API called Entrez [28]. The query was performed using “prenatal nutrition” as a free text keyword (without the quotation marks) in order to take into account the multiple combinations of MeSH terms referring to the subject. Among these 10,000 PMIDs, 2,818 openly accessible papers could be fetched using the BioC API [29]. The reason for the focus on “prenatal nutrition” in this dataset augmentation procedure is because of the second step, where Boguslav et al.’s classifiers trained on prenatal nutrition papers are used.

In the second step, Boguslav et al.’s classifiers are used to annotate the SOIs in our new papers. The reason why classifiers are used instead of annotators is because (1) annotating 2,818 papers (i.e., 715,545 sentences) with experts is unrealistic, and (2) it has been proven by Boguslav et al. that the classifiers had a very good performance on these data. In order to ensure that the classifiers still keep their good performance, we stay as close as possible to the scientific field in Boguslav et al.’s dataset (i.e., prenatal nutrition). Since all ignorance categories are not necessarily interesting for our recommendation setup (e.g., “question answered by this work”, which indicates that the research direction is already tackled in the study in question), a specific subset of ignorance categories are selected (as presented in the Statements of ignorance section): “full unknown”, “explicit question”, “problem or complication”, “future work” and “future prediction”.

In the third step, we gather, for each author in our augmented dataset, the abstract of all papers for which they are first author prior to their oldest paper in our augmented dataset using the OpenAlex API [30]. The rationale is that we want to be able to leverage these abstracts to build a profile of each author prior to what they published in the augmented dataset. Abstracts have the advantage of being generally openly accessible, even when the full papers are not. This renders our technique independent of the open-access status of the author’s papers. The full dataset of abstracts contains 85,342 abstracts.

However, our experiments involve vectorizing our augmented dataset with TF-IDF and such a large dataset does not fit in memory with a reasonable amount of RAM (i.e., more than 20 GB of RAM). In consequence, a methodological subsampling was used in the experiment to make it possible to evaluate the recommendations in different setups. We therefore subsampled, at random, the augmented dataset containing the full papers to 500 unique first authors. This corresponds to 152,189 sentences. Among these sentences, 61,511 were annotated as SOIs and were therefore considered as recommendation candidates.

After subsampling our dataset of full papers, our dataset of abstracts was also subsampled so that it contains the same 500 first authors. In addition, the number of abstracts per author is limited to 5. These 5 abstracts are chosen at random, to avoid any biases. The rationale for selecting 5 abstracts in the experiment is that authors with lots of abstracts (30, 50, or 100, but probably also for 10) are likely to have a well-developed sense of their field and potential research directions. Through this constraint, we, therefore, limited our scope to new researchers. In a real setting, outside the experiment, we would have considered all the author’s abstracts, even if there are more than 5. In the end, the subsampled version of our dataset of abstracts for our experiment contained 1,923 abstracts. The distribution of abstracts per author was the following: 72 authors have 1 abstract, 57 have 2 abstracts, 38 have 3, 42 have 4 and 291 have 5.

Note that our augmented dataset of papers (and its 61,511 SOIs) is exclusively used for testing the recommendations. Indeed, in order to train the methods in our experiments, only the dataset of abstracts is used.

#### Evaluation heuristics

As we cannot easily gather the evaluations of the 500 authors in our dataset to determine the ground truth related to the interestingness of SOIs, we defined heuristics that would allow us to assess the quality of our recommendations. We propose 3 heuristics that are summarized in Table 2. Each of these 3 heuristics (the first-author heuristic, the co-authors heuristic and the concepts heuristic) has pros and cons, so considering them together can provide a more realistic assessment of the recommendation quality. We observed in preliminary experiments that author concepts from tools like OpenAlex are often (1) noisy (i.e., they contain irrelevant concepts for the author) and (2) generic (with concepts such as “computer science”). Because of that, we rely on concepts that can be extracted from the abstracts of the author using a named entity recognition tool. For the concepts heuristic, the concepts in the abstracts and in the SOIs are therefore retrieved using the named entity recognition tool from the work of Raza et al. [31].

**Table 2:** Description of the heuristics used to evaluate the recommendations

Heuristic	Description	Pro	Con
Using the 500 papers’ first authors (we call this heuristic “the first-author heuristic”)	A recommendation is successful for a researcher *r* if, based on the profile of *r* built from the dataset of abstracts, some of the top statements of ignorance recommended to *r* from the augmented dataset in fact come from a paper written by *r*.	Predict the interest of the “next papers” of *r* without having to see them during training.	The quality of the recommendation may partially depend on particular words and expressions *r* uses in their papers.
Using the co-authors of the 500 papers’ first authors (we call this heuristic “the co-authors heuristic”)	A recommendation is successful for a researcher *r* if, based on the profile of *r* built from the dataset of abstracts, some of the top statements of ignorance recommended to *r* from the augmented dataset come from a paper for which the first author is a co-author of *r* in the dataset.	The co-authors’ interests of *r* can be related to the interests of *r* AND the quality of the recommendation does not depend on particular words and expressions *r* uses in their papers.	The co-authors’ interests can be really different than *r*’s interests.
Using the concepts of the 500 papers’ first authors (we call this heuristic “the concepts heuristic”)	A recommendation is successful for a researcher *r* if, based on the profile of *r* built from the dataset of abstracts, some of the top statements of ignorance recommended to *r* from the augmented dataset contains concepts that are also present in the abstracts of *r* in the dataset of abstracts.	Does not depend on the writing style of *r* or of their co-authors.	The quality of the recommendation may rely on a concept in the statement of ignorance that is in fact present in the abstracts of most, if not all, of the 500 authors (i.e., the statement of ignorance would be considered relevant for most, if not all, of the 500 authors).

#### Baseline methods

The recommendation of research directions based on researcher profiles is, to the best of our knowledge, not investigated in the literature. Indeed, the literature on recommender systems for researchers is mainly focused on recommending papers [10, 32], and not specific research directions inside these papers. Furthermore, contrary to our approach, modeling the user profile is generally not performed, as keywords search is proposed instead [32]. As the literature lacks a task like ours, as well as methods and baselines that would come with it, we propose two baselines in this study. The first one is based on BERT and the second one relies on classic machine learning models.

The first baseline sums up researcher profiles using sentence-BERT embeddings [24] (more precisely, “sentence-transformers/all-MiniLM-L6-v2”) on our dataset of abstracts. For this baseline, the embedding of a researcher consists of the average of the embedding of the researchers’ abstracts for which they are the first author. An abstract embedding is defined by the average of all sentence embeddings (given by sentence-BERT) in that abstract. We then also embed the SOIs with sentence-BERT, and the recommendation is provided by the Euclidean distance between the researcher embedding and the SOI embedding (the closer the two embeddings are in the space, the better). This baseline was the most “simple”, yet well-performing, method we could find during our preliminary experiments. In fact, because of the pervasive overfitting issues in this task (briefly discussed in the Discussion section), this simple model was one of the best and outperformed more complex approaches.

The second baseline makes use of more classic machine learning models. In order to have a different representation of the features than the first baseline, we use TF-IDF to vectorize each abstract in the dataset of abstracts. Then, the training phase consists of learning the specific features of each author. In order to do that, we use, for a particular researcher, a classification setup with two classes: whether the abstract belongs to the researcher (i.e., the researcher is the first author) or not. By doing so, the model learns what is specific to the researcher in their abstracts. For the recommendation phase, the trained model is then used on all SOIs and the ones recommended to the author are the ones for which the probability of belonging to the researcher is the highest. The rationale behind this is that if a SOI is considered very close to what the researcher writes in their paper, then it may be a SOI of interest for them.

We noted during our preliminary experiments that, like the first strategy based on sentence-BERT, this last strategy was highly prone to overfitting (see the discussion in the Discussion section). Because of that, more complex models (e.g., neural networks or random forests) yielded worse results in the recommendation phase. Simpler models were systematically better in this setup, as they seem to get rid of the noisy elements in the abstracts (i.e., the textual elements that are not necessary for the recommendation). One model that seemed to outperform the others, because of this overfitting issue, was a Logistic Regression with a Ridge penalty and with C=1. The hyper-parameters of all models were optimized by cross-validation on an external dataset, namely the one of Boguslav et al. [5, 6].

Note that we do not include in the baseline models solutions that rely on keyword searches (like, e.g., the one in the work of Lahav et al. [9]). This is because (1) searching by keywords does not correspond to our setup of modeling users and (2) it is not clear what keywords would best correspond to a researcher to model them. Also note that the proposed heuristics and baseline methods are as much to evaluate RecSOI as to assess the difficulty of our novel task.

## Results

The results for the two baselines and RecSOI, given the three heuristics explained in the Evaluation heuristics section and Table 2, are presented in Table 3. Note that, for each heuristic, a different number of researchers is considered: 500 for the first-author heuristic, 59 for the co-authors heuristic and 496 for the concepts heuristic. Indeed, first, the 500 authors in the first-author heuristic are set by design (we subsampled to have 500 unique first authors in our dataset). Second, concerning the co-authors heuristic, there are only 59 co-authors who are themselves first authors among the 500 first authors. Finally, there are 4 first authors among the 500 for whom there are no concepts in common between the concepts in the SOIs to recommend and the concepts in their abstracts. For these 4 authors, the evaluation of the recommendation quality (according to the concepts heuristic) cannot be computed. As a result, these 4 authors are not considered for this heuristic, which leaves 496 authors to compute the concept heuristic. Note that this has no impact on the use of the recommendation techniques in practice. It only means that for the evaluation of the recommendations in our paper, the concept heuristic cannot be computed for 4 authors.

**Table 3:** Results of the recommendation for a random draw, for the two baselines and for RecSOI according to the heuristics explained in the Evaluation heuristics section and the performance metrics MAP_∃_@5, MAP_∃_@10 and MAP_∃_@20

Metric	Method	First-author heuristic	Co-authors heuristic	Concepts heuristic
MAP_∃_@5	Random	0% $\pm 0.4\%$	0% $\pm 3.1\%$	0% $\pm 0.4\%$
	sentence-BERT	31.2% $\pm 4\%$	**13.6%** $\pm 8.8\%$	65.3% $\pm 4.2\%$
	TF-IDF+LR	23% $\pm 3.7\%$	6.8% $\pm 6.8\%$	43.3% $\pm 4.3\%$
	RecSOI	**34.6%** $\pm 4.2\%$	10.2% $\pm 7.9\%$	**75.6%** $\pm 3.8\%$
MAP_∃_@10	Random	0% $\pm 0.4\%$	0% $\pm 3.1\%$	15.9% $\pm 3.2\%$
	sentence-BERT	37.8% $\pm 4.2\%$	15.3% $\pm 9.2\%$	74.4% $\pm 3.8\%$
	TF-IDF+LR	28.2% $\pm 3.9\%$	8.5% $\pm 7.4\%$	58.1% $\pm 4.3\%$
	RecSOI	**42%** $\pm 4.3\%$	**18.6%** $\pm 9.8\%$	**84.3%** $\pm 3.2\%$
MAP_∃_@20	Random	0% $\pm 0.4\%$	0% $\pm 3.1\%$	46% $\pm 4.4\%$
	sentence-BERT	45.6% $\pm 4.4\%$	**23.7%** $\pm 10.6\%$	82.3% $\pm 3.4\%$
	TF-IDF+LR	38.2% $\pm 4.2\%$	11.9% $\pm 8.3\%$	74.6% $\pm 3.8\%$
	RecSOI	**46.8%** $\pm 4.4\%$	**23.7%** $\pm 10.6\%$	**90.9%** $\pm 2.5\%$

Concerning the evaluation metric, as we want to know the proportion of researchers for which such a recommendation works, we use a score derived from the mean average precision at k (MAP@k). Indeed, while MAP@k is defined as

$$\begin{aligned}{} & {} \text {MAP@k} =\\{} & {} \frac{1}{n} \sum _{r \in R} \frac{\#\ \text {of relevant recommendations for}\ r\ \text {among}\ k}{k}, \end{aligned}$$

where *R* is the set of researchers and *n* is the number of researchers in *R*, we instead use

$$\begin{aligned}{} & {} \text {MAP}_{\exists }\text {@k} =\\{} & {} \frac{1}{n} \sum \limits _{r \in R} \left\{ \begin{array}{ll} 1 &{} \text {if}\ \exists 1\ \text {good recommendation for}\ r\ \text {among}\ k\\ 0 &{} \text {otherwise.} \end{array}\right. \end{aligned}$$

The percentage given by MAP_∃_@k therefore corresponds to the percentage of researchers for which at least one relevant research direction was given in their top k recommendations. In practice, we use MAP_∃_@5, MAP_∃_@10 and MAP_∃_@20 in our experiments. Note that the notion of “good” or “relevant” recommendation is defined by our three heuristics defined in the Evaluation heuristics section.

Confidence intervals are also provided in Table 3. As each percentage in the table is the mean of binary trials (i.e., “Did the researcher get at least one relevant SOI recommended, yes or no?”), the percentages follow a Binomial distribution. The intervals provided in the table are therefore defined accordingly.

In order to assess the quality of the solution when SOIs are drawn at random in the database, the expected random results are also shown in Table 3. Picking at random, in our setup, corresponds to a hypergeometric distribution, as the question is: how many relevant SOIs would I get if I draw *k* SOIs from a large pool of SOIs from which a certain number are relevant for the researcher (depending on the chosen heuristic)? If *k* is the number of SOIs drawn (5, 10, or 20 in our experiments), *n* is the total number of SOIs in the dataset and $n_R$ is the number of SOIs relevant for the researcher, the expected number of relevant SOIs that can be retrieved at random is defined for a hypergeometric distribution as $k*(n_R/n)$. As, in MAP_∃_@k, we consider for each author whether at least one relevant SOI has been found in the top *k* recommendations, “Random” in Table 3 corresponds to the percentage of authors for which $k*(n_R/n) \ge 1$.

### Analysis of the results

One first thing to note when looking at Table 3 is that the problem of finding a relevant SOI, according to the first-author and the co-authors heuristics, is very hard. One can see that the percentage of authors for which $k*(n_R/n) \ge 1$, for $k = 5, 10 \text { and } 20$ is equal to 0% for these two heuristics. This means that for none of the authors, picking *k* SOIs at random lead to an expected number of relevant SOIs retrieved greater or equal to 1. In order to provide a concrete example, let’s consider the author with the median number of SOIs belonging to them in the dataset (i.e., relevance defined by the first-author heuristic), which is $n_R = 99$. Given that the number of SOIs in our dataset is $n = 61,511$, the expected number of relevant SOIs (according to the first-author heuristic) when $k = 5$ SOIs are picked at random is 0.008 relevant SOIs out of a maximum of 5. For $k = 20$ SOIs picked at random, the expected number of relevant SOIs is 0.032 out of a maximum of 20. We are therefore far from having at least 1 relevant SOI for this “median reseacher” when 5 or 20 statements are picked at random. It is therefore not possible to solve the problem by picking SOIs at random.

On another note, by looking at the co-authors heuristic, it seems like recommending SOIs from co-authors’ papers is a very difficult task. Indeed, the best result is a MAP_∃_@20 of 23.7% for sentence-BERT and RecSOI. This may be explained by the fact that, in general, co-authors write very different papers when they are first authors themselves. A potential improvement of this heuristic can therefore be to weight the closeness between author *r* and the papers of their co-authors $r'$ based on the order of the co-authors $r'$ in the papers of *r*. We leave this challenging definition of the co-author heuristic as a future work. But while the scores of the current definition of the heuristic may indicate that this heuristic may not be the best to assess recommendation quality, its results may shed some light on the difficulty of our task.

Another element that is interesting to note is that increasing the number *k* of recommendations mainly benefits the methods that do not perform well to begin with. For instance, for MAP_∃_@20 and the first-author heuristic, the performance increases by only 4.8% for RecSOI, while it increases by 10% for TF-IDF+LR, with respect to the performance for MAP_∃_@10. A similar observation can be made for the concepts heuristic. This seems to indicate that there is a performance saturation for each heuristic. In other words, while methods performing poorly can always do better, correctly recommending starts to become extremely difficult (for a given heuristic) for the remaining percentage of authors.

Finally, we note that if we consider MAP_∃_@5 for the concepts heuristic, the problem can be solved with RecSOI for 75.6% of the authors. In other words, 75.6% of the authors have, in their top 5 recommendations, at least one SOI that share one or several concepts that were found in their abstracts. We also note that this problem is not trivial, as if the SOIs were picked at random, 0% of the authors would get recommendations with a relevant topic.

While the results are high enough for the concepts heuristic and explainably low for the co-author heuristic, it is not clear without further analysis why the results are not higher for the first-author heuristic. In the next section, we aim at clarifying these results and at better understanding these errors.

### Analysis of the first-author heuristic errors

This section aims to analyze why it is hard to obtain better results with the first-author heuristic. Given the description of the first-author heuristic in Table 2, a recommended SOI is relevant for researcher *r*, in the context of our evaluation, if the SOI was in fact written by *r*.

Let’s now consider the worst recommendations according to this heuristic. In order to find them, we consider the authors for which the 5 best-ranked SOIs written by them have the worst ranks for them. This means that, while, ideally, these 5 SOIs written by *r* should be in the top 5 for *r*, they are, for instance, ranked $\sim$10,000 or worse.

One pattern that was identified with this analysis is the “generic SOI issue”. Examples of such issues are shown in Table 4. These SOIs are very generic and do not contain any specific concepts. Because of that, the SOIs cannot be recommended to the authors (e.g., to N. Liu in the table), despite being written by the authors themselves. This kind of SOIs can be observed for different authors for whom the recommendation results were bad (according to the first-author heuristic). The poor performance of the first-author heuristic can be partly explained by the tendency of the recommender to discard generic SOIs, sentences written by the first author but containing few useful concepts, in favor of other SOIs that contain more relevant concepts for the author, but that are written by someone else. One such example of a SOI containing lots of concepts is the following statement from Monk et al. [33] that is recommended to Wei Wu (an author given as example in Table 4): “In addition to these structural abnormalities, biochemical effects include reduced oxidative metabolism in the hippocampus and frontal cortex and altered fatty acid and myelin profiles throughout the brain have been observed.”

**Table 4:** Examples of “generic statements of ignorance (SOIs)” for authors with some of the worst recommendation results

Statement of ignorance (SOI)	Reference to the paper
“The present results are promising enough to support continued investment in this intervention and attempts to improve it”	Liu et al. [34]
“It is urgent to explore effective ways to control this down trend, and research in this area is needed”	Wu et al. [35]
“However, results from randomized controlled trials remain inconclusive”	Harris et al. [36]
“However, its long-term benefits remain to be tested in future studies”	Li et al. [37]
“Therefore, a future large-scale population-based cohort investigation is warranted”	Lin et al. [38]

An insight that can be highlighted by these examples of “generic SOIs” is that SOIs may, by nature, be more frequently generic than claims. While it is very difficult to automatically assess how generic a sentence is, one can argue that scientific claims, by nature, more often state their findings in detail. However, stating something that is unknown or unexpected inherently restricts the possibility of going into details. If this is true, this adds to the intrinsic complexity of the task of recommending research directions based on SOIs, as many SOIs would in fact be written in generic terms, such as in the cases of the authors in Table 4.

One solution to this issue is to consider multi-sentence SOIs. Indeed, thanks to additional sentences, the “generic SOIs” could be contextualized, which could solve the issue. However, this solution suffers from a major drawback: the longer the text representing the statement is, the more difficult it is to adequately embed the concepts inside it. As a result, recommendation performance could suffer. Because of this, embedding statements at the sentence level, as is done in this paper, may be preferable.

This “generic SOI” issue lowers the probability for the author, in the experiment, to be recommended SOIs that they wrote themselves. However, this does not explain why the few specific SOIs written by that author are not recommended to them. We propose two reasons for this. First, it may be that the few SOIs written by the author do not directly relate to their work (e.g., when proposing future works within another field). Second, the few specific SOIs may relate to the current work of the author, but not the previous work used to build their profile. Indeed, let us recall that the abstracts used to build the researchers’ profiles are strictly prior (by construction) to their papers in the dataset of SOIs used for the experiment. This issue should however not be frequent with junior researchers, as their few papers are generally closely related.

If this second hypothesis is true, then this would suggest the relevance of analyzing the changes in researchers’ interests when recommending research directions. To solve this issue, one may try to combine, for instance, RecSOI (our contribution), which is focused on the past, with keyword-based search engines (such as the one of Lahav et al. [9]). While these search engines cannot provide recommendations that match the profile of the researchers based on their past work, as RecSOI does, they can help find interesting directions that are not aligned with the researcher’s past profile.

## Extracting ignorance context

Because RecSOI is based on the extractor of SOIs from Boguslav et al. [5], one strong limitation of our recommender system is that it recommends single sentences only. While this is not an issue for the recommendation algorithm itself (as the context of the sentence is embedded in the sentence-BERT embedding), it can be very difficult for users to know if the recommendations are relevant based on a single sentence only. Indeed, contextual information outside the sentence may be important to understand the future work. This means that, in most cases, users would have to read the paper for each recommended SOI to really know if the statement is relevant for them to pursue or not. In this section, we show and evaluate different ways to provide context to the user.

In order to solve the issue presented above, the Insights on context extraction section will first present our findings on how to provide context to the user. Then, the Evaluation of the usefulness of context section will describe our user study evaluating the usefulness of different ways of providing context.

### Insights on context extraction

When research directions are recommended, the researcher must read the paper containing the research direction to get more information. In some cases, the researcher might realize that they are not interested in pursuing a particular direction. To avoid reading papers of uninteresting directions and to save time for researchers, we propose an analysis related to the extraction of contextual information about SOIs. This task is close to the extractive summarization task.

Providing contextual information about SOIs is not an easy task. Indeed, in many cases, SOIs are not connected to explicit pieces of information in the paper. For instance, some SOIs refer to information that is implicitly absent from, e.g., the experiment. An example of that from Qiu et al. [39] is: “Though consistent with studies of men and non-pregnant women, larger studies that include objective measures of sleep duration, quality and apnea are needed to obtain more precise estimates of observed associations.” The implicit information behind this SOI is that apnea was not really measured in the authors’ experiments (only if the participants were snoring), making the association of apnea with other measures difficult to objectively establish. This information, however, is not explicitly present in the paper and is implicitly inferred by the reader after reading the paper and the SOI.

When information about a SOI is explicitly provided, however, the relevant pieces of information are generally in the vicinity of the SOI. Indeed, more often than not, the sentences that immediately precede the SOI provide the necessary context to understand the statement. The problem, therefore, becomes “what are the passages in the SOI’s paragraph that contain enough contextual information?”

During our preliminary experiments, the most powerful methods to solve this problem were large language models (LLMs). More specifically, we observed that the results of available open-source LLMs did not compare with LLMs such as GPT. In the next section, we show some results from GPT to solve that problem and propose an experiment to quantitatively assess the usefulness of LLMs with respect to naive heuristics.

### Evaluation of the usefulness of context

One way to quickly grasp the context of a SOI is to provide the paragraph that contains that statement. This section evaluates how often it is the case that providing the paragraph is useful to understand the SOI. On top of that, because paragraphs can sometimes be lengthy, we also evaluate when highlighting shorter passages within the paragraph is helpful. In order to evaluate the usefulness of highlighting, we use both a simple heuristic (highlighting the sentence before the SOI) and a more complex solution (using prompt engineering to tune GPT-3.5 [40] to provide relevant highlights in the paragraph).

To present our evaluation and the corresponding results, this section comprises three parts. The Experimental setup section first explains the overall experimental setup. Then, the GPT prompt engineering and other LLMs section digs deeper into the prompt engineering phase that led to the results of GPT-3.5 in the experiment. This section also extends to other LLMs and their results. Finally, our results are reported and analyzed in the Results and analysis section.

#### Experimental setup

The dataset used for this experiment is a subset of Boguslav et al.’s dataset [5, 6]. Because the purpose of this experiment is to assess if the additional context is useful to better understand the SOI, and not to assess if the SOI is indeed about ignorance, a manually annotated dataset is used. Furthermore, to focus the evaluator’s attention on the context rather than on the SOI itself, only SOIs that explicitly stated future work were selected. With this selection, we expect that the evaluators will focus on whether the context helps understand what the future direction is about and not how to use the SOI as a research direction.

**Fig. 2:**
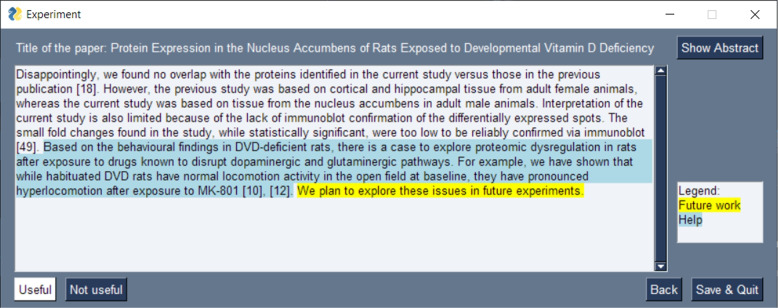
Main panel of the interface used for the experiment about context. The paragraph (from McGrath et al. [41], in this example) in which the future work sentence (in yellow) is mentioned is provided. In this example, the blue highlight corresponds to the important contextual sentences according to GPT-3.5

The interface is composed of two panels: a main panel to gather the evaluation of the evaluators and a secondary panel to get some optional comments. The main panel of the interface used for the evaluation can be seen in Fig. 2. In this main panel, the evaluator can see the SOI (highlighted in yellow), the paragraph surrounding this statement, and some blue highlights depending on the strategy. The question for each SOI was: “Is this paragraph and its potentially highlighted part(s) useful for you to understand what this statement of ignorance is about?” While waiting for an answer (“Useful” or “Not Useful”), the interface records the time it takes for the evaluator to give their answer. In this main panel, the abstract of the paper containing the SOI is accessible by clicking on a button. Each time a decision, “Useful” or “Not Useful”, is made by the evaluator, the secondary panel opens, asking whether the evaluator has any comment regarding the decision they just made. For each evaluator, the evaluation ends when a “Useful” or “Not Useful” decision has been provided for all SOIs.

The experiments are built upon 30 SOIs selected at random. Each SOI is presented three times during the experiment: once with its contextual paragraph but no highlight, once with the previous sentence highlighted in the paragraph, and once with highlights provided by GPT in the paragraph. This resulted in 90 “Useful”/“Not Useful” trials to perform per participant. The strategies behind the highlights were not provided to the participants (i.e., they only saw highlights, without knowing what generated them). Each participant received the trials in a different order. The randomization was designed by block: for each SOI, each of the three highlight strategies is randomly assigned to one of three blocks. The SOIs inside each block are then further shuffled. This ensures that the same statements with two highlight strategies are not close to each other in the experiment. A further condition was added to filter and only keep the randomized orders where at least five trials separate two highlight strategies on the same SOIs. This condition is necessary because, in some rare cases, two highlight strategies for the same SOI can be assigned at the end of a block and the beginning of the next block. In that case, the same SOI (but with different highlight strategies) would be seen twice in a row, which would bias the experiment as the evaluator would remember their previous judgment when making the second one.

The eight evaluators are all researchers in bioinformatics or have a strong knowledge of biology. This ensures that (1) as they are all researchers, they have a good understanding of what constitutes a future work statement in a research paper, and (2) they have sufficient background to understand the biomedical papers in our dataset. The experiment took between around 60 and 90 minutes, depending on the participant.

In the next section, we discuss in greater length the use of LLMs to provide the highlighted parts of our experiment.

#### GPT prompt engineering and other LLMs

After some prompt engineering, we discovered that developing a very complex prompt was not necessary to obtain good results on our extraction task. The prompt that worked the best with GPT-3.5 was the following:

Given the following paragraph from a scientific paper:

“{PARAGRAPH}”

Please provide the most relevant passage(s) from this paragraph that can help a researcher understand “{STATEMENT OF IGNORANCE}" in the paragraph and that is not “{STATEMENT OF IGNORANCE}" itself. Please do not add anything other than the passage(s) in your response.

with {PARAGRAPH} being the paragraph that contains the SOI referred by {STATEMENT OF IGNORANCE}. GPT-4 [42] offered similar results on our task for a much greater cost. Given the lack of difference in the results, GPT-3.5 is considered for the whole experiment.

Other LLMs, particularly open-source ones like BLOOM [43], have been tested on our task with different prompts. Unfortunately, none could rival the performance of GPT-3.5. Common issues were (1) not sticking exactly to (i.e., modifying) the text of the paper and (2) adding additional, non-requested information. For the worst LLMs on our task, the outputs were not relevant.

#### Results and analysis

Table 5 contains the preferences of each participant, where a preference of a given highlight strategy A over B is defined by the fact that the participant found A useful but not B for the same future work statement and the same paragraph. Each row in the table provides the percentage of time, for the same paragraph, a participant preferred a certain combination of methods (with GPT highlights (“GPT” in the table), with the previous sentence highlighted (“PS” in the table), and the paragraph without any highlight (“Paragraph” in the table)). For instance, P1 having 10% for “Both GPT and PS” means that for 10% of the provided future work statements, P1 considered that highlighting using GPT and highlighting using the previous sentence in the paragraph was useful, but having the paragraph only without highlights was not useful.

**Table 5:** For each participant, the percentage of time (over 30 paragraphs) each combination of contextual strategies has been deemed useful on the same paragraph. PS stands for the “previous sentence strategy” and Paragraph for “no highlight, the paragraph only is provided”

What is Useful?	P1	P2	P3	P4	P5	P6	P7	P8
Everything	53.3%	43.3%	53.3%	43.3%	36.7%	26.7%	43.3%	36.7%
GPT Only	6.7%	6.7%	0%	0%	0%	6.7%	6.7%	13.3%
PS Only	10%	3.3%	0%	0%	3.3%	10%	3.3%	13.3%
Paragraph Only	0%	3.3%	6.7%	0%	20%	6.7%	3.3%	0%
Both GPT & PS	10%	23.3%	3.3%	3.3%	3.3%	13.3%	6.7%	3.3%
Both GPT & Paragraph	13.3%	13.3%	30%	10%	10%	13.3%	16.7%	0%
Both PS & Paragraph	6.7%	0%	0%	0%	16.7%	0%	10%	30%
Nothing	0%	6.7%	6.7%	43.3%	10%	23.3%	10%	3.3%

A paired t-test analysis over each pair of methods and all participants shows that there is no one-fits-all solution. Indeed, the participants can be clustered in groups of preferences, which are canceling out when considered all together. This signals that a more detailed analysis must be performed, in particular, to identify clusters among participants.

One first thing to note is that while the preferences for methods are spread differently among the participants, considering everything useful (first row called “Everything” in Table 5) is always the most frequent option. In the most extreme case, P1 and P3 consider that everything is useful (all the possibilities, i.e., the paragraph without highlight, with the previous sentence highlighted and with GPT’s highlights) more than half of the time.

A second thing to note is that the case where nothing is useful (last row called “Nothing” in Table 5), always has a low percentage of preference (except for P4). In other words, presenting something alongside the future work statement (the paragraph with or without highlight) was almost always useful for the participants (100% of the time for P1, 83.3% for P2 and P3, 56.7% for P4, 90% for P5 and P7, 76.7% for P6 and 96.7% for P8).

Another trend that has been observed is that participants tend to always prefer GPT highlights (“GPT Only” line in the table), no highlight (“Paragraph Only” line in the table), or both (“Both GPT & Paragraph” line in the table), over naively highlighting the previous sentence (“Paragraph Only” line in the table). Indeed, it can be seen in Table 6 that P2 and P4 consider that GPT is significantly more useful than the previous sentence (“PS”), P5 considers that providing the paragraph without highlights (“Paragraph”) is more useful than the other options, and P3 considers that both GPT and no highlight (“Paragraph”) are better than highlighting the previous sentence (“PS”) only. This result is expected, as it means the sentence before the future work statement is not always related to the future work in question. In these cases, it is better to either use a smarter strategy (e.g., GPT) or to not provide anything at all.

**Table 6:** Table representing the methods that were significantly preferred (*p*-value threshold of 0.05) by each participant. Participants with * have a significant preference with a *p*-value threshold of 0.1

	GPT	PS	Paragraph
Over GPT		P8	P5, P8
Over PS	P2*, P3, P4*		P3, P5
Over Paragraph	P2		

As the most frequent case, for all participants, is when everything is useful, the usefulness of highlights when the paragraph without highlights is considered useful was analyzed (see Table 7). The purpose of this analysis is to assess the usefulness of highlights when the participants detected useful information in the paragraph when there were no highlights. In that case, when the paragraph without highlights is considered useful, all participants considered that highlighting the previous sentence was not useful. This indicates that when the participants can identify the useful information in the paragraph, highlighting the previous sentence is not useful for them. This is more rarely the case for GPT, where only P5, P7, and P8 significantly considered that GPT’s highlights were not useful when the paragraph alone is useful.

**Table 7:** Table representing the methods that were significantly preferred (*p*-value threshold of 0.05) by each participant when the paragraph is considered *useful* by the participant. Participants with * have a significant preference with a *p*-value threshold of 0.1

	GPT	PS	Paragraph
Over GPT		P8	P5, P7, P8
Over PS	P2, P3, P4*, P6		P1, P2, P3, P4*, P5, P6, P7, P8
Over Paragraph			

Another interesting insight comes from the opposite case, i.e., when the paragraph is considered not useful by the participants (see Table 8). A paragraph without highlights can be considered not useful for two reasons: (1) the paragraph does not contain useful information to understand the future work statement, or (2) the useful information in the paragraph is hidden in noise and, because of that, the participant did not see the useful information. Five participants considered that it is significantly useful to provide highlights in this context: P1, P2, and P6 considering that any way to highlight is useful in that case, while P7 has a preference for highlights provided by GPT, in this context, and P8 has a preference for highlighting the previous sentence.

**Table 8:** Table representing the methods that were significantly preferred (*p*-value threshold of 0.05) by each participant when the paragraph is considered *not useful* by the participant. Participants with * have a significant preference with a *p*-value threshold of 0.1

	GPT	PS	Paragraph
Over GPT		P8	
Over PS			
Over Paragraph	P1, P2, P6, P7	P1, P2, P6, P7*, P8	

Several things can be concluded from this analysis. First, the future work statement should always be shown embedded in the paragraph in which it appears, as it is very rare that this is not useful (see the last row of Table 5). Second, as there is a possibility that the useful information in the paragraph has been missed by the researcher, and as providing highlights rarely hurts (see the row “Paragraph Only” in Table 5 to see the percentage of time providing the paragraph has been considered useful, but not the highlights), some highlights should be proposed with the paragraph containing the future work statement. Finally, these highlights should come from an advanced method (such as GPT in our study) instead of a naive one. However, what our study also shows is that even a very advanced way to provide highlights (such as using one of the best-performing LLMs) can have difficulties to compete with a situation where no highlights are provided. This means that, in order to make the highlights useful, they should be provided by a high-performing method that can identify the pieces of information that may be hidden in the paragraph and that may help understand the future work statement.

## Discussion

Several elements of discussion arise from our study of recommending research directions. First, we discuss the different ways to embed researcher profiles. Second, using interpretable models made it possible to highlight interesting insights when solving the task. Third, the difficulty of recommending research directions based on researcher profiles is discussed. Fourth, we mention different fairness issues that can arise from such a recommendation of research directions. Fifth, as no study is empty of limitations, we discuss the limitations of our study in order to suggest future work. Finally, we sum up the significance of our work for the scientific community as a whole.

### On the different ways to embed researcher profiles

Many different elements can be considered to embed researcher profiles when recommending research directions. Indeed, in addition to a summary of the previous abstracts (that we perform with sentence-BERT), here are other elements that can be taken into account: embedding summary of the whole previous papers, concepts retrieved in previous abstracts or papers by a concept recognizer, concepts in co-author abstracts or papers, concepts related to the papers cited by the papers of the author, etc.

Each of these strategies has pros and cons. For instance, considering whole papers to represent a researcher (instead of abstracts only) can provide more information but can also bury the important information in a mass of irrelevant texts. Furthermore, alternative strategies can be of interest in other setups than the one considered in this paper. For instance, if no abstract or paper is available for the researcher (for instance, because they are very new researchers), then recent information (abstracts, papers, and/or concepts) about the researcher’s supervisor can be used.

### What can be learned from the interpretable models?

Interpretable models are models that provide users access to their inner workings [44, 45]. Examples of interpretable models are sparse linear models, for which the weights can be extracted and studied, and decision trees with their human-friendly representation. As we use interpretable models in our experiments, like linear models with TF-IDF vectorization as features, we can leverage the information they provide about their modeling of the data and the task to get new insights. In fact, our interpretable models show that models easily overfit when performing the recommendation. Despite this issue, the weights of linear models can provide important clues about the reasons for this overfitting issue.

An analysis of the interpretable models shows that recommender systems can choose spurious features. For instance, if a researcher often generates a certain typo or refers to a specific city, then a SOI containing this typo or city may be used by the model as an important feature for the recommendation to this researcher. This, of course, leads to poor performance during the recommendation phase. Therefore, the simpler the model is, the less likely it is to overfit terms specific to the author that are in fact irrelevant to the recommendation.

However, these interpretable models also show that recommendations can sometimes be correctly performed with a combination of a few concepts that would otherwise be buried in long texts.

### On the difficulty of the task

The results from our experiment in the Evaluation section show that the task at hand is in fact very difficult. This is partly due to SOIs that are very generic.

Indeed, if we consider for instance the first-author heuristic, a recommendation is considered good for a researcher *r*, in our experiments, if the recommended SOI has in fact been written by *r*. However, if a particular SOI written by *r* is so generic that it is of no interest to the researchers in the field, then the probability of having it in the top 5 recommendations for *r* themselves is very low. Furthermore, if *r* tends to write all of their SOIs in such a way, then it may be that none of the SOIs written by *r* can in fact be recommended to *r*, which would participate in a bad result according to the first-author heuristic. The heuristic based on concepts alleviates this issue, as any SOI that contains concepts also present in the abstracts of *r* is considered a relevant recommendation candidate.

One other solution to avoid this issue, that we leave as a future work, is to gather experts in the field covered by the dataset of SOIs (e.g., in our case, experts in prenatal nutrition) and to ask them the question, “would this researcher be interested to work on at least one of these 5 SOIs?”. This solution however requires the gathered experts to study the researchers in the dataset in order to know their work and to be able to judge if the recommendations can be relevant for the researcher or not.

### Possible fairness issues in the recommendation of research directions

While recommending research directions can make it easier for junior researchers to navigate their field, possible fairness issues can also arise. In this section, we highlight these potential fairness issues in order to raise awareness and inspire future work on the subject.

There are three categories of persons that are usually considered targets of fairness issues [46]: consumers, producers, and subjects. Consumers are the users of the recommender system, which corresponds, in our case, to the researchers using our system to obtain recommendations for research directions. Producers, on the other hand, are the persons producing the elements that are recommended. In our case, these persons are the authors of the SOIs and, therefore, of the papers containing them. Finally, the subjects are the persons concerned by the studies in these SOIs. For instance, if a SOI states that additional studies are required about a certain disease in a certain population, this population can also be the target of unfairness.

One first consumer fairness issue relates to the researchers who are non-native English speakers. Indeed, the more the sentences in their abstracts deviate from ordinary English phrasing, the more difficult it can be to match the researcher’s embedding to the SOI embeddings. A second consumer population that can be the target of unfairness is the most junior researchers. Indeed, these researchers may not yet use, in their few papers, the vocabulary of the field following the common usage that is well-known by more senior researchers. For these two issues related to an under-represented use of the language, fine-tuning the sentence-BERT embedding model with examples of non-native and junior researchers can be a solution. Finally, authors working on niche subjects can also suffer from unfairness. However, while this last point also deserves attention, it can more easily be tackled, as it is done by our proposed method RecSOI. Indeed, a niche research direction will be recommended by RecSOI as long as the SOI about this niche subject is close to at least one abstract of the researcher. However, RecSOI relies on an embedding model trained on data that may not contain lots of documents about the niche subject. Because of that, the resulting embeddings of sentences about niche subjects may be of lower quality, which can therefore lower the recommendation performance for niche subjects.

The fairness issues related to producers mirror the ones related to consumers. Indeed, some SOIs may be less often recommended and therefore proposed as research directions, if they have been written by non-native English speakers or junior researchers, or if they state ignorance about a niche subject. This means that the work of these researchers is less likely to be used as a basis for future work. As for consumer issues, fine-tuning the sentence-BERT embeddings to obtain embeddings that are equally good for non-conventional research sentences in English can be a solution.

However, the populations that may be the most impacted by the fact that some SOIs may be less recommended are the subjects in the related studies. Indeed, if, for instance, the medical aspects of a population from a certain non-English speaking country are almost exclusively studied by researchers from that country, then medical abnormalities and other research directions related to this population will be less recommended. However, if the producer unfairness issues mentioned above are solved, and all SOIs are all equally good for recommended, then this fairness issue may be solved at the same time.

### Limitations of this work

Like all studies, our work comes with a set of limitations that are important to consider. First of all, our dataset is focused on prenatal nutrition papers. While additional work on generalization will need to be conducted, it is important to note that it is difficult to gather 500 or more authors from the literature to assess recommendations made for them. Likewise, gathering senior researchers to read researcher profiles and assess recommendations is also very difficult. This is why focusing on a specific field makes it easier to develop classifiers with good performance to automatically annotate the SOIs from a large set of papers.

However, in addition to its focus on a specific field, our work is also focused on sentences. Indeed, SOIs are considered to be contained in sentences in our work and the work of Boguslav et al. [5, 6]. However, in some cases, multiple sentences are needed to describe the ignorance comprehensively. While we leave the detection and the recommendation of multi-sentence SOIs as a future work, it is worth noting that it can make the task even more difficult, as more words and concepts would have to be encoded in an embedding.

Another limitation of our work, which would require further studies and a dedicated solution, is that we consider SOIs without knowing if the statements have already been answered in recent papers. This task of determining if a solution to the problem has been provided is very difficult for many reasons. Some of these reasons are that (1) the words and the level of formalization used in the paper containing the problem and the one with the solution may be different, and (2) the proposed solutions are generally not complete answers to the question: they make specific hypotheses, have limitations, etc. This makes it therefore very hard to automatically solve the problem “Is this lack of knowledge, not a lack of knowledge at all anymore?”.

Next comes a limitation that is specific to our novel task and its solution: we implicitly make the hypothesis that the papers containing the research directions to recommend are freely and openly accessible. This is certainly not always the case, but research directions inside papers can hardly be extracted if the papers are not accessible. One solution to this issue is to propose research directions as we do for openly accessible papers and recommend whole papers based on meta-data when the papers are not accessible. See Haruna et al. [20] for a solution to recommend papers when meta-data only are available.

### Significance of this work

Despite the inherent limitations and the need for future exploration, our study’s findings can prove useful far beyond our dataset on prenatal nutrition and the broader scope of biomedical research. Our work lies in the larger spectrum of how we keep track of what we know as well as recognize what we have yet to discover. Such an approach is particularly crucial given the accelerating pace of scientific output [1]. We believe that our study is one of the first to propose a systematic method to counter the decline in innovation, disruptiveness, and return on scientific investments [2–4]. By providing a structured approach to understanding and organizing existing knowledge, the task and the system we propose could be of great utility to other scientific fields, promoting efficient navigation through extensive literature and assisting in the identification of under-explored areas.

## Conclusion

This paper introduced a new task - recommending research directions based on statements of ignorance (SOIs) - and a system to solve it. While many papers in the literature focus on recommending scientific papers, our work goes further by recommending specific sentences in these papers that can lead to new research directions. While the mass of scientific papers grows bigger and bigger, we believe it is important to develop solutions to navigate this mass. This is especially true for junior researchers who do not know yet all the potential directions in their field.

Our solution, RecSOI (Recommender of research directions using Statements Of Ignorance) leverages weighted BERT-like embeddings of previous abstracts to build researcher profiles. These profiles can then be used to find SOIs that are relevant to them. Different heuristics are used to estimate the relevancy of our work. For the concepts heuristic, we show that RecSOI achieves a MAP_∃_@5 of 77.2%. This means that 77.2% of the authors have, in their top 5, at least one SOI that contains at least one concept present in their abstracts.

Furthermore, as one of the contributions of this paper is the task itself, we also provide a detailed discussion of its uses and limitations. Among the important elements that are discussed, we enumerate potential fairness issues that can arise when dealing with this task.

Our work opens the door to many different avenues of future work. One of the most important future work avenues is to detect if a stated lack of knowledge is not a lack of knowledge anymore in the literature. This requires, for a specific SOI, browsing the literature in order to find if a paper fully answers the stated ignorance. While this is a very hard problem, we believe it is one of the most important in the field of “science of science”.

Other future work relates more closely to the solution brought in this paper. First, a multi-sentence extraction of SOIs can be developed and then used for recommendation. Second, a more sophisticated metric learning procedure that does not fall into the overfitting trap can be developed to build researcher profiles. This can achieved in two different ways: (1) by defining a metric that would consider the different sentence-BERT dimensions without sticking too closely to the training data; and/or (2) by defining a new way to embed the researcher profiles, so that metrics applied to these profiles would not overfit. Another interesting future work would also be to add a name disambiguation module when extracting past abstracts, in order to make sure that the author of the abstracts is indeed the author for which recommendations are requested. Another way to build researcher profiles, and an interesting future work, would be to determine the abstract from the past of the researcher that are still relevant to characterize their current research. Finally, a cross-field recommender system can be developed. Indeed, one can argue that the expertise and interests of some researchers can cross scientific domains (e.g., a machine learning researcher following a research direction from the field of AI law).

Through the contributions of this paper, we aim to help science overcome one of its largest current challenges: helping researchers find research directions that are relevant to them.

## Data Availability

The annotated dataset of Boguslav et al. is available at https://github.com/UCDenver-ccp/Ignorance-Question-Work-Full-Corpus. RecSOI code, as well as the related data and resources, are available at https://github.com/AdrienBibal/RecSOI. Additional papers can freely be accessed with the PubMed Entrez API, using the query “prenatal nutrition” (without quotes). To get information about the authors, their co-authors and their papers, the OpenAlex API can freely be used.

## Abbreviations

LLMs: Large language models
MAP@k: Mean average precision at k
MAP@_∃_k: Mean average precision at k with at least one relevant recommendation
PMID: PubMed ID
PS: Previous sentence
RecSOI: Recommender of research directions using statements of ignorance
SOI: Statement of ignorance
